# The Shipwrecked Fishermen and Mariners' Benevolent Society

**Published:** 1894-02-24

**Authors:** 


### Special Appeals.

### THE SHIPWRECKED FISHERMEN AND MARINERS' ROYAL BENEVOLENT SOCIETY.

The appalling results of the late terrific gales, in conjunction with the too probable further sea disasters of a very stormy winter, will beyond doubt have excited, just now, no little public concern as to what medium is nationally available for coping with all the consequent maritime distress.

This highly opportune moment, therefore, is officially taken to explain to the country at large that, for over half a century past, in comprehensively organised manner, there has existed, and been utilised for every known occasion of maritime need throughout the kingdom, this national medium of the corporation of the Shipwrecked Mariners' Society.

With a central executive, meeting weekly in London, and responsible local representatives, armed with due powers of instant action, at every port, fishing town, or other station, numbering altogether some 1,000, this great maritime relief organisation is directly on the spot everywhere, ready, on the broadest basis of Christian charity, to befriend and to aid without failure or delay.

Under this wide and appropriately national scope the shipwrecked sailor, wherever landed, is, in his forlorn condition, at once taken up, clothed, fed, medically tended, and sent free to his home; while the bereaved dependents of the drowned, in their sudden desolation, are immediately sought out and their necessities met, with both primary and secondary succour, in money and in kind.

Besides these chief sufferers the Shipwrecked Mariners' Society embraces within its relief functions the constant cases of individual distress to seafarers of all grades through the many accidents and disabilities incidental to their perilous calling, no applicant with genuine tale of woe being ever cast adrift hopeless or unassisted.

That self-same work for the nation which the Shipwrecked Mariners' Society has thus daily, and even hourly, carried on in the past it has already been doing, is at this moment doing, and will continue still to do, with the good help of the public in connection with the present unparalleled casualties and losses of life as well as with those of the future.

But liberal as from time to time has been the public support accorded to the Shipwrecked Mariners' Society, that support has indisputably too far fallen short of what would be and should be the essential amount as compared with the ever-increasing magnitude and exigency of the requirements.

The pressing exceptional demands of this disastrous moment make it now urgent to crave that it may be publicly remembered with the prompt recognition of a generous sympathy that the Shipwrecked Mariners' Society's national cause is that truly of humanity and of charity alike, and that its national claims are those in very deed of the nation's mariners and fishermen themselves, their widows, and their orphans.

(Signed, on behalf of the Central Executive), E. S. Adeane, Vice-Admiral, Chairman of the Committee of Management; David Mainland, Chairman of the Finance Committee; W. R. Buck, Secretary.

